# An integrated engineering worldview of synthetic biology education through the lens of webinar based pedagogy

**DOI:** 10.3389/fbioe.2024.1431374

**Published:** 2024-10-01

**Authors:** Iain George, Paul Ross, Yixian Yang, Martin Holub, Nina Rajpurohit, Ibrahim Aldulijan, Jacob Beal, Alejandro Vignoni, Dennis Mishler

**Affiliations:** ^1^ Analytical Performance Center, Danone North America, Louisville, CO, United States; ^2^ BioStrat Marketing, Boynton Beach, FL, United States; ^3^ School of Biomedical, Nutritional and Sport Sciences, Faculty of Medical Sciences, Newcastle University, Newcastle UponTyne, United Kingdom; ^4^ Delft University of Technology, Delft, Netherlands; ^5^ Lieber Institute for Brain Development, Johns Hopkins University, Baltimore, MD, United States; ^6^ Systems Engineering, Stevens Institute of Technology, Hoboken, NJ, United States; ^7^ Intelligent Software & Systems, Raytheon BBN Technologies, Cambridge, MA, United States; ^8^ Synthetic Biology and Biosystems Control Lab, Instituto de Automatica e Informatica Industrial, Universitat Politecnica de Valencia, Valencia, Spain; ^9^ Department of Molecular Biosciences, Center for Systems and Synthetic Biology, The University of Texas at Austin, Austin, TX, United States; ^10^ The Freshman Research Initiative, College of Natural Sciences, The University of Texas at Austin, Austin, TX, United States

**Keywords:** iGEM, DBTL, global education, webinar education, synthetic biology education

## Abstract

Synthetic biology is an interdisciplinary field that brings together engineering and biology concepts alongside the arts and social sciences to develop solutions to pressing problems in our world. The education of students entering this field has relied on a diverse set of pedagogical methods to accomplish this goal. One non-profit group, iGEM–the International Genetically Engineered Machine competition, has been a driver of students’ awareness of synthetic biology for the last 20 years giving many young researchers their first experience in the field of synthetic biology. Dissemination of synthetic biology concepts by iGEM has occurred through several programs including a webinar series started during the 2020 COVID pandemic. The iGEM webinar series successfully engaged students by taking inspiration from synthetic biology programs in Europe, North America, and Asia that had themselves evolved alongside iGEM. The webinar designers modeled the content after their experiences in iGEM as well as their academic courses, pedagogy, and mentoring experiences. This series has produced globally accessible pedagogy for both technical synthetic biology knowledge and the communication skills necessary to build and communicate synthetic biology projects. The hope is that this series functions as a lasting blueprint that can be used by future educators in synthetic biology and other disciplines to reduce barriers that students face when attempting to enter cutting edge fields.

## 1 Introduction

Synthetic biology education, reflecting the field it represents, draws inspiration, and adopts ideas from disciplines of Science, Technology, Engineering, Arts, and Mathematics (STEAM) while integrating relevant aspects of these disciplines into a cohesive pedagogical framework (Cameron et al., 2014; Hallinan et al., 2019). These interdisciplinary educational philosophies challenge students to be not just bench-side technicians, but thoughtful researchers and engineers who are capable of interrogating and assessing synthetic biology’s impact on their communities (Simons, 2020). This broader scope has necessitated a range of pedagogical methods to effectively educate synthetic biology practitioners and prepare them for the expanded role they are expected to play (Diep et al., 2020; Steel et al., 2019; Wachter et al., 2022).

In the field of synthetic biology many of the pedagogical methods that have proven effective over time trace their origin to the iGEM (International Genetic Engineering Machine) competition (Dy et al., 2019; Kelwick et al., 2015). iGEM is a non-profit organization dedicated to providing high school and university students the opportunity to learn about synthetic biology by providing them a platform they can use to develop their research and engineering skills while addressing global and local problems. However, iGEM also provides students with stated objectives and a timeline that forces them to engage in activities that are dual use: research and educational. The timeline comes to an end each year with an annual conference or the “iGEM Jamboree,” where teams of students present the results of their year-long synthetic biology projects. While developing their projects for the Jamboree, students must: conduct human practices - gaining a broader understanding of their research problem and proposed solution, engage other young scientists and engineers - building the synthetic biology community, and engage experts in the form of judges - establishing the importance of standards and experimental rigor. Additionally, students learn about and apply many of the basic methodologies that are core to synthetic biology. One specific example is the application of the engineering Design, Build, Test and Learn (DBTL) cycle (Lawson et al., 2019; Stock and Gorochowski, 2024). The DBTL cycle lends structure to an iterative learning process that, in the iGEM context, guides students in designing genetic parts, creating biological systems, and testing system effectiveness in the lab. The learning students gain from the outcomes reinforces the importance of the next iteration of the cycle and recasts failure as an integral step of learning and conducting research. Using this iterative approach, students gain valuable hands-on experience and insight into the frameworks and complexities of synthetic biology research and improve their problem-solving abilities.

Since its inception, iGEM has strived to make synthetic biology more accessible to students around the world. This includes endeavors such as the distribution kit of DNA parts (iGEM) (Smolke, 2009), free DNA synthesis through corporate sponsors, and the inter-lab study (Beal et al., 2018; Beal et al., 2021). However, not all teams have the resources, be it money, space, or mentorship, to fully utilize these offerings, potentially limiting their engagement and growth through iGEM participation. This was particularly true during the 2020–2021 COVID pandemic, when labs across the world were closed to undergraduate researchers. In response to this, the iGEM Measurement Committee (now the iGEM Engineering Committee) created a synthetic biology webinar series focused around the iGEM competition timeline and objectives. The webinar series served two purposes: 1) a way to replace those lost educational experiences of being in a lab conducting research, and 2) a means to give a wider group of students access to synthetic biology pedagogy. The series makes introductory synthetic biology content readily and freely available to anyone with internet access both synchronously and asynchronously. It delivers instructions on how to use synthetic biology research tools and techniques, such as *in silico* design of genetic circuits, various cloning strategies, and modelling. Overall, it serves as an example of how to make synthetic biology knowledge globally accessible, lowering barriers to entry and generating pedagogy that can be used by anyone.

## 2 iGEM webinar origins and pedagogy

The iGEM webinar series was the outcome of an *ad hoc* arrangement made by members of the iGEM Measurement Committee in response to the 2020 COVID pandemic. This group included faculty members, graduate students, and other researchers in academia and industry. The content and structure of the webinar series reflected their collective educational and research expertise. This included their pedagogical experiences from their respective institutions and regions, primarily academic labs in Europe, North America, and Asia. These individuals mostly came from programs that evolved in areas with strong structural support for synthetic biology and iGEM, such as Canada, the Netherlands, and the United Kingdom as well as from regions where support is more *ad hoc*, such as the United States. The designers modeled the content after their experiences in iGEM as well as their academic courses, pedagogy, and mentoring experiences. The pedagogical content included project ideation, the various wet lab and dry lab techniques needed to conduct research, and eventually how to communicate one’s research to the iGEM scientific community. A syllabus for the webinar series is provided in Table 1 with explanation of the key components of each webinar.

**TABLE 1:** Syllabus for the iGEM Webinar series.

Topic	Type	Webinars
Project Ideation • What makes a good project? • How to brainstorm a project	Soft Skills	• iGEM Project Ideation and Overview (2021) ^a^
Getting Started on an iGEM Project • What to consider now that you have an idea • Identification of relevant genes to use in your project	Soft Skills	• Getting Started (2021) ^a^
Modelling and Design of Genetic Circuits • Ordinary Differential Equations (ODE) • Analysis of genetic circuits dynamics. Mass action kinetics and Hill functions (Design) • Feedforward and feedback network motif • Model parameter estimation from experimental data (Learn) • Applying these concepts to different genetic circuits as case-studies	Dry Lab	• Modelling for Synthetic Biology - iGEM 2020 Opening Weekend Festival (2020) • Modeling I: ODEs and Hill Functions (2021) ^a^ • Modeling II: Modeling Circuits with ODEs and Experimental Data (2021) ^a^ • Modeling for SynBio: from ODEs to Gene Expression (2022)
Basic Molecular Biology and the Designing of DNA Circuits • Synthetic biology and key concepts in molecular biology • What are DNA parts and considerations in building complex genetic circuits • Usage of bioinformatics in sequence design	Dry Lab	• Basic Molecular Biology and Designing DNA Sequences (2021) ^a^ • Design of Biological Networks (2021) • Bioinformatics - Computational Approaches to Analyze Sequences (2021)
DNA Cloning and Assembly Strategies • Overview of assembly methods • BioBrick, Golden Gate, Gibson and Modular (MoClo) assembly methods • Transformation and sequencing confirming	Wet Lab	• DNA Assembly Strategies (2021) • DNA Assembly Techniques (2020) • Gibson Assembly and Yeast (2020) • Transformation and Sanger Sequencing (2020)
Advanced Synthetic Biology Lab Techniques • Cell free systems for gene expression • Genome engineering tools including CRISPR/Cas9 • Lab automation tools	Wet Lab and Dry Lab	• Cell-Free Systems (2020) • Introduction to CRISPR (2020) • Genome Engineering (2021) • Lab Automation (2021)
Characterization of Genetic Circuits • Expression of proteins and basic characterization • Using standards in fluorescent measurements. Plate reader and flow cytometer calibration • Quantifying genetic circuit output using fluorescence	Wet Lab	• Protein Purification and Characterization (2020) • Quantifying Fluorescence and Cell Count with Plate Readers (2021) ^a^ • Quantifying Fluorescence and Cell Phenotypes with Flow Cytometry (2021) ^a^
Demonstrating and Communication of Your Project • How to demonstrate the work from an iGEM project • Effective ways to communicate results	Soft Skills	• Demonstrating Your Project’s Successes (2021) • Communicating your iGEM Project (2021)

^a^ The year the webinar was presented is indicated in parentheses. Webinars given in both 2020 and 2021 are listed as 2021.

The inspiration for the webinar’s structure and content came from many sources and content creators. Below, we highlight one example from the University of Texas at Austin where already-existing educational curriculum inspired some of the webinar content and structure. At the University of Texas at Austin, the iGEM team has been closely affiliated with the Freshman Research Initiative (FRI) program (Rodenbusch et al., 2016), which emphasizes experiential learning through students conducting research as part of their academic course work. During the first semester, students learn basic lab techniques alongside the concepts required to conduct synthetic biology research and to initiate new research projects, in a structure similar to how the iGEM webinar series was designed. After the first semester, students conduct research under the guidance of a faculty member, gaining ownership over their project (Corwin et al., 2018). For the UT Austin iGEM team, this has meant that as the students conduct project ideation, they begin to learn the requisite skills and concepts that are needed for the entire year. The FRI program and UT Austin iGEM team also rely heavily on peer mentors (previous students) and sometimes graduate students or postdoctoral fellows for training first year students. This has allowed the UT Austin team to develop successful projects and from that broader institutional support. Ultimately, the webinar series aims to inspire the implementation of synthetic biology pedagogy and programs globally and we hope students engaging in the webinars may seek to reform their local curriculum to develop future centers of synthetic biology research and education.

**FIGURE 1:**
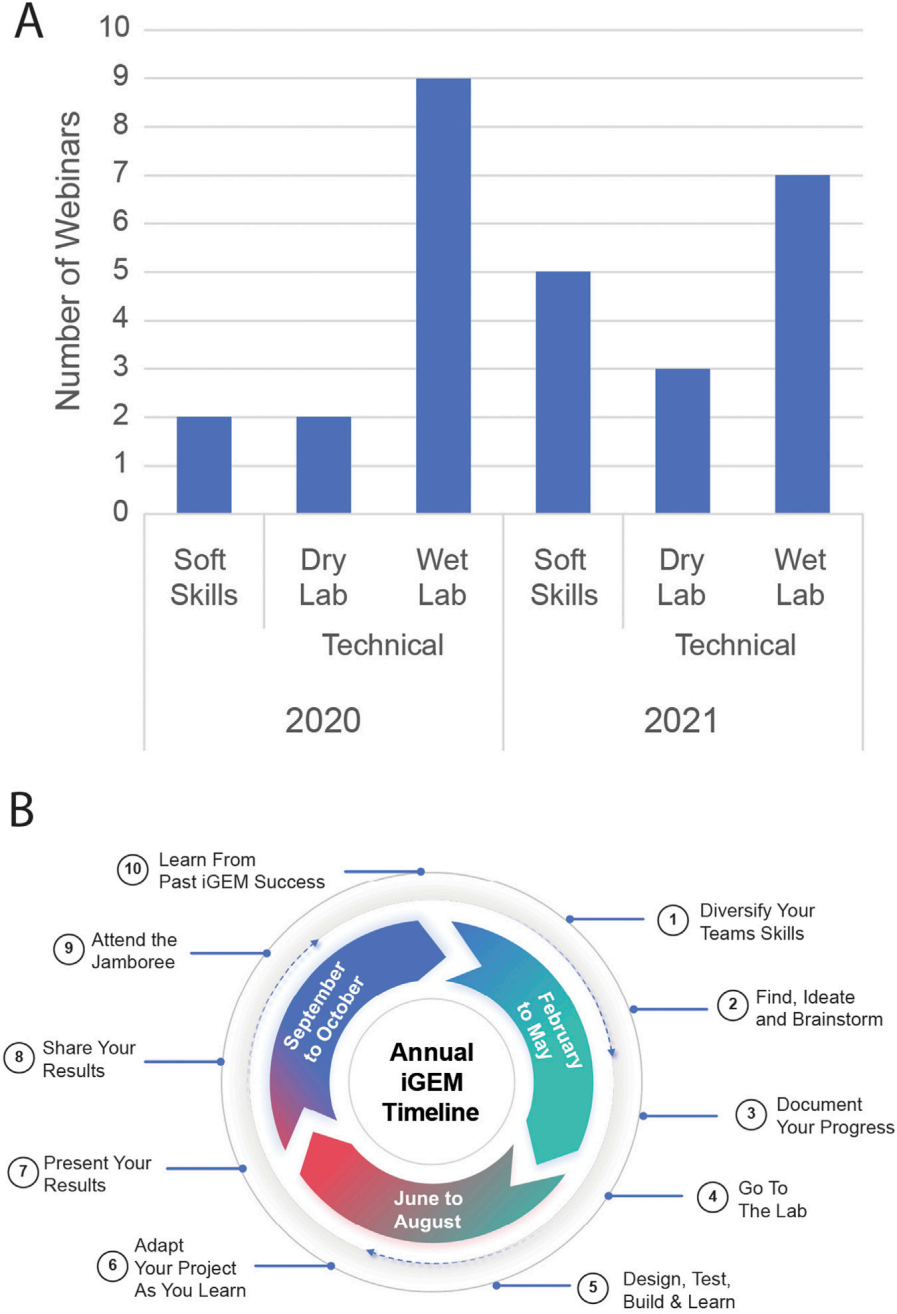
Overview of the webinar topic categorization and timeline for the 2020 and 2021 series years. **(A)** Soft skills represent webinars focused on project ideation and presentation, while technical includes core scientific topics relevant in the project. **(B)** The generalized timeline for completing different goals in the iGEM competition. This shows early brainstorming and context finding as key parts of developing a project for the season. Adapted and reproduced with permission from iGEM.

The programming that forms the webinar series is curated for an audience of young researchers and introduces topics ranging from soft skills to technical focus (Figure 1A). Technical synthetic biology methods formed the majority of topics and included *in silico* modeling, genetic circuit design, cloning, genome engineering, and genetic circuit characterization, whose mastery is essential for gaining an understanding of the field. The webinars also touched upon soft skills that are crucial to student success including project ideation and the ability to communicate one’s work to a broad audience. Together, these topics combined into a curriculum that followed the annual iGEM project cycle, beginning with ideation and brainstorming (Figure 1B).

### 2.1 Project ideation and brainstorming

Bridging the gap from a blank page to a plan for implementation represents a significant challenge for teams competing in iGEM. The webinar series focused on brainstorming and ideation in 2021 with a simple goal, to equip students with the tools, techniques, and methods they could use to ideate and structure their projects as they prepared to enter the iGEM season (Figure 1B).

The ideation session focused on brainstorming, introducing participants to a structured approach they could draw on for guidance and a strategy for proceeding when faced with a blank page. The approach consisted of three steps: self-guided reflection, exchanging ideas with other members of the team and soliciting ideas from external stakeholders through extensive consultation. This process exploits the iterating interplay of these steps to refine the student’s ideas and present them in a more concrete and useable form. In the case of iGEM, this allows teams to not only start to target technical challenges, but also to address how their proposed ideas have the potential to impact stakeholders.

To illustrate the ideation methods, several examples of previous iGEM projects were included in the webinar. These case examples were used to demonstrate how a “good project” could be both conceived and implemented. Each was used as an opportunity to understand how the iterative cycles of ideation could arrive at a project that was both viable for the iGEM season and how this research connects to stakeholders. This process exemplifies the goal of the webinar series, by providing a venue to transmit key concepts and filling in gaps with structure in the early phases of their projects.

### 2.2 Technical training and experience

Mastering the technical skills used in synthetic biology grants students the ability to effectively design, build, and test their ideas in both the wet and dry lab. This is a common aim of synthetic biology pedagogy programs that focus on the practical applications of the concepts being taught. Recognizing this, the 2020 and 2021 webinar series focused 21 of 28 sessions on technical topics and the concepts underlying them (Figure 1).

Topics included basic and advanced molecular biology techniques, describing how to design experiments and the considerations that should be taken to have the best chance at success. The wet lab topics included gene cloning strategies and featured many of the techniques needed to carry out these types of experiments. Participants were led through examples that described how to obtain DNA sequences for a gene of interest followed by how to modify the sequence and how to design specific DNA primers. Students then learned how PCR could be used to amplify out these regions or when it would make sense to consider DNA synthesis to obtain gene fragments. The webinars also explored various assembly strategies, including traditional BioBrick assembly, Type IIS assembly, and Gibson assembly (Knight, 2003; Engler et al., 2008; Gibson et al., 2009). As part of this, the webinar creators discussed the advantages and caveats of each method. The webinars next focused on how to use the assembled DNA by transforming them into cells where the function of these genes could be characterized through different methods of measurement. A large variety of measurement methods were covered from fluorescence systems such as plate readers and flow cytometry to protein purification and characterization.

The webinars also covered dry lab topics for modelling and *in silico* experiments (i.e., computational simulations) of biological systems behavior. These seminars aimed to give young researchers a crash course in how to use these tools to make their design so that it would later help inform their wet lab work. The seminars also stressed how the outcomes of their experimental tests could feed back into these models, further supporting their learning and projects. Topics explored included Hill functions and the effects of different regulatory elements in genetic constructs, such as activators and repressors. Additionally, students learned how predictions could be made around networks of genes in a bacterial cell, depending on how it had been designed, with the demonstrations working from a simple genetic circuit to a complex multifactorial genetic circuit.

The topics covered in the wet lab technical webinars provided young researchers with a stronger base knowledge of specific core synthetic biology techniques. This was expected to fill in gaps and function as a replacement for missed lab work due to the pandemic or for teams that had yet to establish wet lab practices in their region. Although ostensibly focused on the iGEM competition, much of the technical content is applicable to anyone approaching research in synthetic biology or related disciplines for the first time.

### 2.3 Documentation and presentation of findings

A common challenge in research is how to document the work and turn that into a narrative that can be effectively transmitted to others. Tufte described several “Principles of Graphical Excellence” including “Graphical excellence is the well-designed presentation of interesting data–a matter of substance, of statistics and of design” (Tufte, 2001). This is an important consideration for iGEM teams given the complexity that scientific research presents. After the 2020 webinar year, we realized there was a need to better describe methods for how students could communicate and present their findings. As a result, two webinars were added in the fall of 2021 that addressed how a team could communicate their project and successes.

In “Demonstrating your Project’s Success,” students were introduced to storytelling and how they could use this to share their message. This involved exploring what goes into building an effective and engaging story for the audience, including the constraints that the presenters would have such as time available, in-person versus remote delivery, the diversity of the audience’s expertise and the visuals to present. Also presented were specific examples of good visual design that can clearly and concisely aid in telling the student’s story.

Similarly, the “Communicating Your Project” webinar expanded upon the ideas of how to demonstrate success using iGEM project case studies. Specific stories were taken from the Leiden and DTU-Denmark iGEM teams that had been effective in the simplicity and directness of the language used to describe their work and the impact. As part of this analysis, we examined specific parts of each project to understand how they had arrived at the visuals or language that described their project. We also discussed areas for improvement to demonstrate that these stories are not perfect and have areas for continuous improvement. Lastly, we examined examples of project documentation by looking at how successful teams had detailed their work on the iGEM registry and wiki pages.

The demonstration and communication webinars allowed students to gain a better toolset for building out their documentation and presenting their story. This set of webinars was presented during the fall run-up to the Jamboree where students would be able to actively use the presenting skills they had explored in this webinar. There were multiple takeaways but two included, first teaching students how to produce simple, well-documented figures with language that could be shared with a broad audience. The second was building communication skills that would enable students to be effective in sharing their project’s stories. These skills are critical components beyond the competition, as they enable students to be effective science communicators to a broad range of audience that they will interact with throughout their careers.

## 3 Global implementation and cross-pollination

Synthetic biology education programs do not operate in isolation. This is mirrored in the field of synthetic biology where prior work through supporting groups has encouraged the exchange of ideas (Moon, 2022). This has been enabled through groups such as the US Synthetic Biology Research Engineering Center (SynBERC) and the European Union Horizon Europe program that were established to provide funding for collaborative efforts in the design of synthetic biology tools (Donati et al., 2022; van der Vlugt, 2020). Through these funding mechanisms, a collaborative and cross-pollinating environment in the field of synthetic biology has been enabled. The iGEM webinar series expands this collaboration idea beyond basic research and into a globally accessible effort that has helped transcend borders to enable access to synthetic biology pedagogy.

How could global synthetic biology pedagogy (described above) flow into an undergraduate course? As an example of convergent evolution, Zhejiang University near Shanghai, China serves as a framework for how we envision webinar content could be combined with wet and dry lab training. This school runs a synthetic biology class with a similar structure to the iGEM webinar series, where theoretical concepts of synthetic biology are taught in tandem with technical components needed to carry out a project (Yang and Yang, 2022). This course is accelerated, occurring over 8 weeks, with the program providing 3 weeks of lecture-based classroom instruction in synthetic biology, a week of project ideation, 3 weeks of hands-on practical application of the theoretical principles in a teaching laboratory and a week of summary and presentation of work. By supplementing theoretical pedagogy with practical application, Zhejiang’s synthetic biology curriculum ensures that students have practical hands-on experience beyond their traditional lecture-based experiences in synthetic biology. This example shows how an institution could leverage the structure of the iGEM webinar series and build upon it with hands-on practical application time in the lab to give students a strong understanding of both theoretical and practical synthetic biology practices. For future groups, we envision them using the webinar content in conjunction with hands-on lab research activities, mirroring the Zhejiang University example presented here to produce a cohesive synthetic biology education program.

**FIGURE 2:**
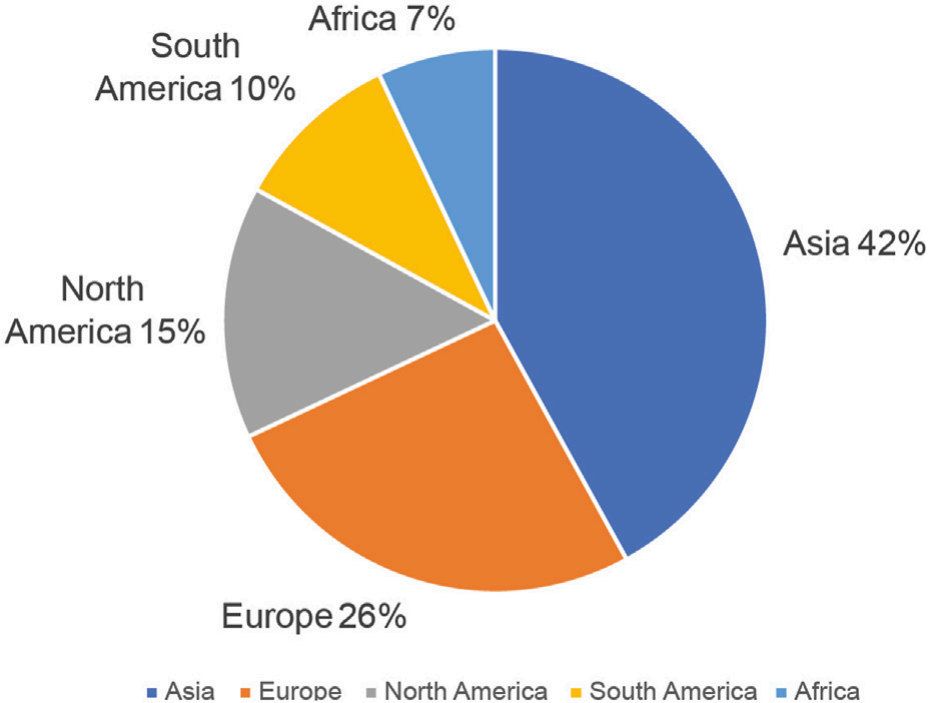
The distribution of participants by geographical region as a percentage over the series run in 2021.

The webinars’ virtual format and uploading to YouTube has helped break down barriers and enhance inclusivity by making material broadly available that would previously have been accessible only to those with a university affiliation. Over time, the number of participants in the webinars series has expanded to encompass participants from institutions representing more than 40 countries and virtually all regions. This is demonstrated in the global audience (Figure 2) of more than 500 participants during the live sessions participating from all parts of the world. Since posting these webinars on asynchronous platforms, they have been viewed over 6,000 times on iGEM’s Video Universe and over 129,000 times on YouTube (iGEM Engineering Committee, 2021). The view count of these videos continues to grow year over year indicating the continued value of the content both to iGEM and the broader community. Anecdotal evidence gleaned from the feedback of participants points to the value and impact that the series delivered both for their project and their own development. The following statement is representative of the feedback solicited: *“For me, the most valuable webinar was the one on Modeling because it helped me understand how much a project’s success depends on developing a good model. The insights our team got from the webinar were useful because they helped us figure out which model to use for the topic our team was researching, use derivatives to fit the model to the experimental data, and make use of Matlab software to evaluate the data we collected and refine the model.”* (comments given by Yixian Yan in 2024). We used participant feedback such as this to revise and improve the webinar series in terms of content offered and how the series was structured. Through this content, young researchers benefit from global cross-pollination of ideas and pedagogical methods as this material is introduced into their local institutions.

Concerning accessibility of the webinars, spoken and written language barriers remain an impediment to the transmission of any educational material. While the webinar series was presented in English, it has been made available in other languages through YouTube’s artificial intelligence-based closed captioning functions. Furthermore, the iGEM Engineering Committee comprises a large cohort of members who represent a diverse range of languages and educational backgrounds and have dedicated themselves to answering questions posed by students in their native languages throughout the world. This has been particularly true for South American teams who have actively engaged the committee for content in Spanish.

From a pedagogy perspective, iGEM stands out as a global interdisciplinary collaboration and cross-pollinating hub for synthetic biology concepts. The webinar series functions as a global resource, bringing students together in the study of key synthetic biology concepts because of the international nature of iGEM and the cross-pollination that iGEM enables, providing a global integrated worldview of synthetic biology. This manifests through interactions and transmission of ideas from these different regions, enabling connections that would otherwise not have a geographical justification for their existence. Concerted efforts to reach out to areas with more limited access to scientific knowledge remains a valuable and noble goal of this program. Continued development of pedagogy on a global scale is a fundamental aspiration and an opportunity to evolve and improve education of synthetic biology concepts.

## 4 Conclusion

Synthetic biology education is evolving rapidly in response to the transformation of a field that is becoming more technically mature, socially oriented, and globally represented. iGEM is a major global cross-pollinator in this field and brings together students from a wide geographic space while providing students their first practical experience with research in the field of synthetic biology. This has enabled iGEM to be an incubating space for globally accessible synthetic biology pedagogy, with a webinar series started at the beginning of the pandemic being a major thrust. With a broad-based curriculum that encompasses instruction in scientific principles, technical skills, and communication, the webinar series has been aligned with the cross disciplinary focus at the core of synthetic biology. Although ostensibly focused on the iGEM competition, this pedagogical content is applicable to anyone approaching research in synthetic biology or related disciplines for the first time. This pedagogical content is intended to aid existing synthetic biology programs as well as lower the barriers of entry for regions new to the field. The webinar series encourages students to be more than technicians at the bench. The hope is that this webinar series functions as a lasting blueprint for synthetic biology pedagogy that can be used to train future generations of synthetic biologists.

## Data Availability

The original contributions presented in the study are included in the article/supplementary material, further inquiries can be directed to the corresponding authors.

## References

[B1] Beal J. Baldwin G. S. Farny N. G. Gershater M. Haddock-Angelli T. Buckley-Taylor R. (2021). Comparative analysis of three studies measuring fluorescence from engineered bacterial genetic constructs. PLOS One. - 16, e0252263. 10.1371/journal.pone.0252263 34097703 PMC8183995

[B2] Beal J. Haddock-Angelli T. Baldwin G. Gershater M. Dwijayanti A. Storch M. (2018). Quantification of bacterial fluorescence using independent calibrants. PLOS One 13, e0199432. 10.1371/journal.pone.0199432 29928012 PMC6013168

[B3] Cameron D. E. Bashor Caleb J. Collins J. J. (2014). A brief history of synthetic biology. Nat. Rev. Microbiol. 12 (5), 381–390. 10.1038/nrmicro3239 24686414

[B4] Engler C. Kandzia R. Marillonnet S. (2008). Precision cloning method with high throughput capability. PLOS ONE. 10.1371/journal.pone.0003647 PMC2574415 18985154

[B5] Corwin L. A. Runyon C. R. Ghanem E. Sandy M. Clark G. Palmer G. C. (2018). Effects of discovery, iteration, and collaboration in laboratory courses on undergraduates’ research career intentions fully mediated by student ownership. CBE - Life Sci. Educ. 17, ar20. 10.1187/cbe.17-07-0141 29749845 PMC5998318

[B17] Diep P. Boucinha A. Yeung B. A. Kell B. Chen X. Tsyplenkov D. (2020). Challenges in undergraduate synthetic biology training: insights from a Canadian iGEM student perspective. bioRxiv. 10.1101/2020.11.04.365999 34237221

[B26] Donati S. Barbier I. García-Soriano D. A. Grasso S. Handal-Marquez P. Malcı K. (2022). Synthetic biology in Europe: current community landscape and future perspectives. Biotechnol. Notes, 3–31. 10.1016/j.biotno.2022.07.003 PMC11446344 39416454

[B6] Dy A. J. Aurand E. R. Friedman D. C. (2019). YouTube resources for synthetic biology education. Synth. Biol. 4 (1), ysz022. 10.1093/synbio/ysz022 PMC7445784 32995544

[B8] Gibson D. G. Young L. Chuang R. Y. Venter J. C. Hutchison C. A. III Smith H. O. (2009). Enzymatic assembly of DNA molecules up to several hundred kilobases. Nat. Methods 6, 343–345. 10.1038/nmeth.1318 19363495

[B9] Hallinan J. S. Wipat A. Kitney R. Woods S. Taylor K. Goñi‐Moreno A. (2021). Future‐proofing synthetic biology: educating the next generation. Eng. Biol. 3, 25–31. 10.1049/enb.2019.0001

[B11] Kelwick R. Bowater L. Yeoman K. H. Bowater R. P. (2015). Promoting microbiology education through the iGEM synthetic biology competition. FEMS Microbiol. Lett. 16, fnv129. 10.1093/femsle/fnv129 26260156

[B12] Knight T. (2003). Idempotent vector design for standard assembly of biobricks. Available at: https://dspace.mit.edu/handle/1721.1/21168 Accessed 27 September, 2024).

[B13] Lawson C. E. Harcombe W. R. Hatzenpichler R. Lindemann S. R. Löffler F. E. O’Malley M. A. (2019). Common principles and best practices for engineering microbiomes. Nat. Rev. Microbiol. 17, 725–741. 10.1038/s41579-019-0255-9 31548653 PMC8323346

[B14] Leiden University (2024). Molecular genetics and biotechnology (MSc) extra-curricular. Available at: https://www.universiteitleiden.nl/en/education/study-programmes/master/biology/molecular-genetics-and-biotechnology/about-the-programme/extra-curricular Accessed April 28, 2024).

[B16] Moon H. (2022). iGEM 2021: A Year in Review. BioDesign Res., 2022. 10.34133/2022/9794609 PMC10521691 37850126

[B18] Rodenbusch S. E. Hernandez P. R. Simmons S. L. Dolan E. L. (2016). Early engagement in course-based research increases graduation rates and completion of science, engineering, and Mathematics degrees. Eng. Math. Degrees CBE-Life Sci. Educ. 15, ar20. 10.1187/cbe.16-03-0117 PMC4909342 27252296

[B19] Simons M (2020). The diversity of engineering in synthetic biology. Nanoethics 14, 71–91. 10.1007/s11569-019-00348-1

[B20] Smolke C. D. (2009). Building outside of the box: iGEM and the BioBricks foundation. Nat. Biotechnol. 27, 1099–1102. 10.1038/nbt1209-1099 20010584

[B10] Steel J. J. Bates K. L. Barnhart M. D. (2019). Investing in our nation’s future military leaders’ synthetic biology knowledge to understand and recognize threats and applications. Synth. Biol. 4–1. 10.1093/synbio/ysz024 PMC7445786 33033745

[B15] Stock M. Gorochowski T. E. (2024). Open-endedness in synthetic biology: A route to continual innovation for biological design. Sci Adv. 10 (3). 10.1126/sciadv.adi3621 PMC11809665 38241375

[B22] The Economist Moon landing apart (2024). Moon landing apart, Indian science punches far below its weight. The Economist. Available at: https://www.economist.com/science-and-technology/2024/01/03/moon-landing-apart-indian-science-punches-far-below-its-weight.

[B23] Tufte E. (2001). The visual display of quantitative information. Cheshire, Connecticut: Graphics Press LLC, 51.

[B24] van der Vlugt C. J. B. (2020). Horizon scan of synthetic biology developments for microorganisms with application in the agri. Food Sect. EFSA Support. Publ. 3. 10.2903/sp.efsa.2020.en-1664

[B25] Wachter G. K. A. Gallup O. Bayne J. Horsfall L. (2022). Synthetic biology landscape in the UK. Biotechnol. Notes 3, 70–74. 10.1016/j.biotno.2022.07.002 PMC11446367 39416441

[B7] Yang F. Yang J. (2022). Design and implementation of the course on Synthetic Biology based on the concept of general education. Chin. J. Biotechnol. 68 (4). 10.13345/j.cjb.210582 35470633

